# The emerging role of minor intron splicing in neurological disorders

**DOI:** 10.15698/cst2018.03.126

**Published:** 2018-02-22

**Authors:** Daniel Jutzi, Maureen V. Akinyi, Jonas Mechtersheimer, Mikko J. Frilander, Marc-David Ruepp

**Affiliations:** 1 Department of Chemistry and Biochemistry, University of Bern, CH-3012 Bern, Switzerland.; 2 Graduate School for Cellular and Biomedical Sciences, University of Bern, CH-3012 Bern, Switzerland.; 3 Institute of Biotechnology, University of Helsinki, FI-00014, Finland.; 4 United Kingdom Dementia Research Institute Centre, Maurice Wohl Clinical Neuroscience Institute, Institute of Psychiatry, Psychology and Neuroscience, King’s College London, SE5 9NU London, UK.

**Keywords:** minor spliceosome, pre-mRNA splicing, neurodegeneration, ALS, SMA, FUS, TDP-43

## Abstract

Pre-mRNA splicing is an essential step in eukaryotic gene expression. Mutations in *cis*-acting sequence elements within pre-mRNA molecules or *trans*-acting factors involved in pre-mRNA processing have both been linked to splicing dysfunction that give rise to a large number of human diseases. These mutations typically affect the major splicing pathway, which excises more than 99% of all introns in humans. However, approximately 700-800 human introns feature divergent intron consensus sequences at their 5' and 3' ends and are recognized by a separate pre-mRNA processing machinery denoted as the minor spliceosome. This spliceosome has been studied less than its major counterpart, but has received increasing attention during the last few years as a novel pathomechanistic player on the stage in neurodevelopmental and neurodegenerative diseases. Here, we review the current knowledge on minor spliceosome function and discuss its potential pathomechanistic role and impact in neurodegeneration.

## INTRODUCTION

Pre-mRNA splicing is an essential step in eukaryotic gene expression. It is predominantly a co-transcriptional process during which the non-coding introns are excised from precursor mRNA (pre-mRNA) molecules and the flanking exons are joined (spliced) together resulting in translation-competent mature mRNA molecules. In most metazoan organisms, pre-mRNA splicing is carried out by two separate spliceosomes that function in parallel, specializing in distinct intron types 1. The bulk of the introns are removed by the major (U2-dependent) spliceosome and feature, in addition to the nearly invariant GT-AG sequences, relatively divergent consensus sequences in their 5' and 3' termini. This group constitutes approximately 99.5% of all introns that are collectively called major or U2-type introns. Additionally, the minor (U12-dependent) spliceosome excises a small subset of introns that contain highly conserved 5' splice sites (5'ss) and branch point sequences (BPS) 2,3. These minor or U12-type introns are found in approximately 700-800 genes in humans and represent approximately 0.5% of all human introns 4. The U12-type introns coexist with the U2-type introns in the same genes. Typical minor intron containing genes contain one, but occasionally two or three U12-type introns and multiple U2-type introns 3,5. The positions of the U12-type introns within their host genes are evolutionarily conserved, not only within vertebrates, but in some cases also in invertebrates 6.

The key difference between the two machineries is in the composition of the small nuclear ribonucleoproteins (snRNPs), and at a functional level, in the initial intron recognition steps. Both machineries are composed of five small nuclear RNAs (snRNAs) that associate with a large number of protein components to make up snRNPs 7,8. Of the five snRNAs four are unique to each spliceosome (**Table 1**). Specifically, the major spliceosome is composed of U1, U2, U4 and U6 unique snRNAs, while the respective snRNAs in the minor spliceosome are U11, U12, U4atac and U6atac. U5 snRNA is shared between the two spliceosomes. In either spliceosome the U4 and U6 as well as their functional analogues U4atac and U6atac snRNPs form a trimeric structure with the U5 snRNP called U4/U6.U5 or U4atac/U6atac.U5 tri-snRNP, respectively. In these complexes U4 and U6 or U4atac and U6atac snRNAs are extensively base-paired with each other 9,10. Similar higher order organization is found between U11 and U12 of the minor spliceosome, which form a U11/12 di-snRNP 11,12 while the respective U1 and U2 snRNPs of the major spliceosome exist as mono-snRNPs.

**Table 1:** Table 1. Major vs Minor spliceosome snRNAs and associated proteins. **+**Sm proteins**-**B/B’, D1, D2, D3, E, F, and G *U5 snRNA is shared between major and minor spliceosomes **# **Denotes the centrifugal fraction in which the proteins were identified **Note:** Dynamic changes in protein compositions of the snRNPs during splicing stages are not shown and the reader is referred to 13,14 for more details.

**Spliceosome**	**snRNAs**	**Core associated proteins**	**References**
**Major**	U1	Sm proteins^+^, U1-A , U1-C, U1-70K	4 13 15 16 17
	U2	Sm proteins^+^, **12S**^#^: U2-A’, U2-B’’, 17S^#^: SF3a and SF3b complexes, hPrp43	13 16 17 18 19
	U5*	Sm proteins^+^, **20S**^#^: 52K, 40K, hPrp8, hBrr2, Snu114, hPrp6, hPrp28, hDib1	13 20 21
	U4/U6	Sm proteins^+^, LSm proteins2-8, **13S**^#^: CypH, 15.5K, hPrp3, hPrp31, hPrp4	13 14 22 23
**Minor**	U11/U12	Sm proteins^+^, **18S**^#^: SF3b complex, 20K (*ZMAT5)*, 25K (*SNRNP25*),31K (*ZCRB1*)	
		35K (*SNRNP35*), 48K (*SNRNP48*)*,* 59K *(PDCD7*) 65K (*RNPC3*),Urp (*ZRSR2*)	24 25 26
	U4atac/U6atac	Share proteins with U4/U6 snRNAs of the major spliceosome	22

In contrast to the divergent snRNA composition, most of the protein components are thought to be shared between the two systems. All snRNPs except U6 and U6atac are associated with a seven-membered ring of Sm-proteins necessary for snRNP function and biogenesis 27. Conversely, U6 and U6atac snRNPs contain seven Lsm proteins 28. Furthermore, all specific proteins associated with either tri-snRNP are thought to be identical between the two systems 22. Differences in protein composition are associated with U11/U12 di-snRNP in comparison to U1 and U2 in the major spliceosome. Specifically, U11/U12 di-snRNP contains seven unique protein components not found from the major spliceosome (see **Table 1**) 24,25.

**Figure 1:**
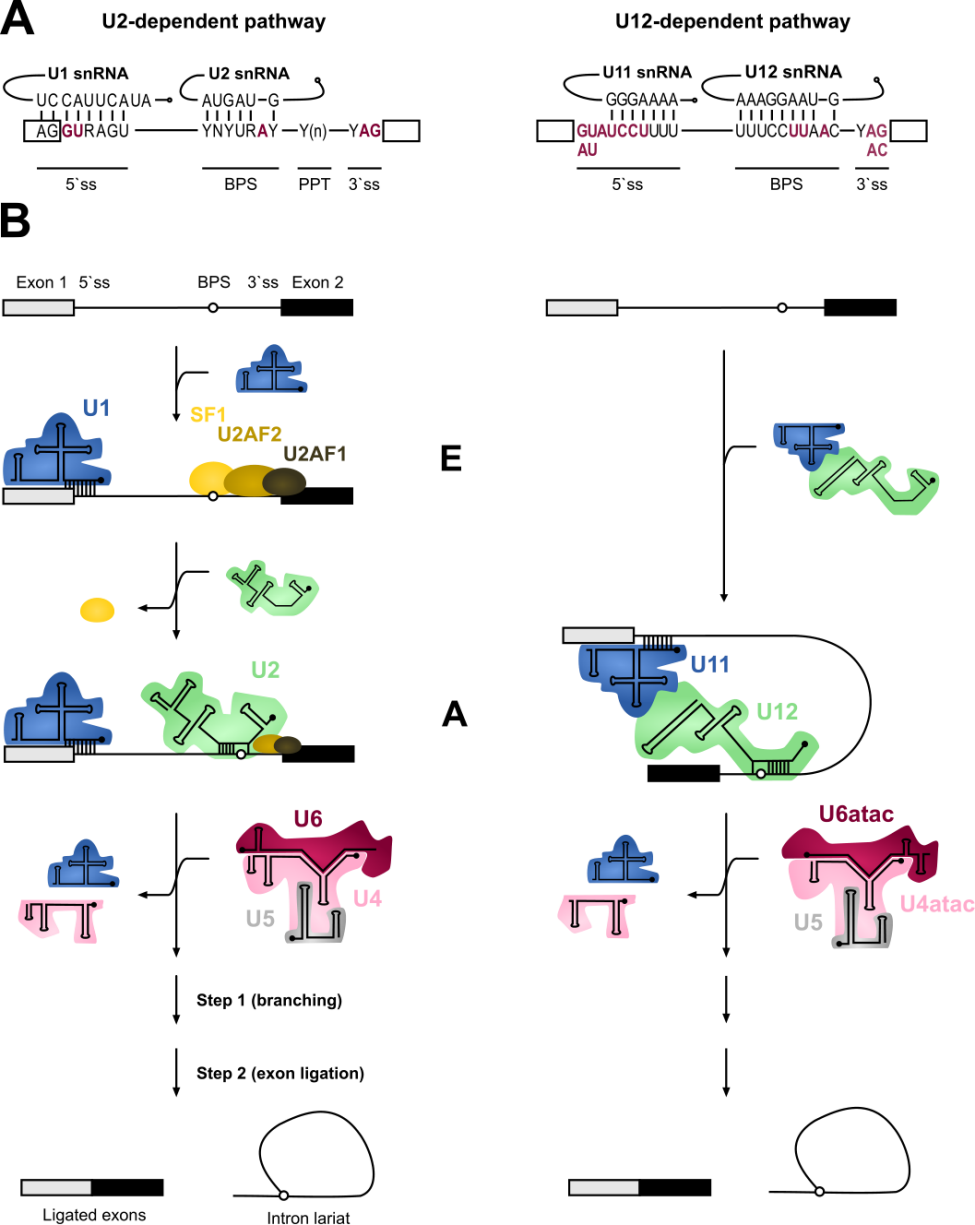
FIGURE 1: Major versus minor intron splicing. **(A)** Major (U2-type) and minor (U12-type) introns differ in their *cis*-acting 5'ss and BPS elements. The (nearly) invariant nucleotides are highlighted in red letters. Non-coloured letters indicate a clear preference for a nucleotide at a given position and potential base parings with the respective snRNAs are depicted. Base modifications of snRNAs are omitted. Minor introns are subdivided into AT-AC or GT-AG minor introns based on their terminal dinucleotides. **(B)** Major and minor introns are recognized differently by their respective spliceosomes, which assemble on their substrates in a stepwise manner. Major introns are initially recognized by the U1 snRNP binding to the 5’ splice site, SF1 binding to the branch point sequence (BPS) and U2AF2/1 heterodimer recognizing the polypyrimidine tract (PPT) and the 3’ terminal AG dinucleotide, respectively. Recognition of the BPS by the U2 snRNA displaces the SF1 and converts the E complex to complex A. In contrast to the major introns, 5’ss and BPS of minor introns are recognized cooperatively by U11 and U12 of the di-snRNP, respectively, thereby forming the minor intron A complex. The subsequent steps in the splicing process are very similar between the two systems and intron recognition is followed by the association of major and minor tri-snRNPs, respectively, giving rise to (presumably) similar catalytic structures and catalytic steps of splicing.

Both the U1 and U2 and the analogous U11/U12 function in the initial recognition of introns and it is this step of splicing where the two systems show most differences. In addition to the significant differences in the extent of U11/5'ss vs U1/5'ss and U12/BPS vs U2/BPS base-pairing (**Figure 1**), the main difference is in the mechanism of intron recognition. With major introns, the individual U1 and U2 snRNPs independently bind to the 5’ss and BPS sequences, respectively. Additionally, the U2AF1/2 protein dimer recognizes a polypyrimidine track (PPT) found upstream of the 3’ splice site (3’ss) as well as the terminal AG dinucleotide in major but not minor introns. In contrast, the recognition of the 5’ss and BPS of minor introns takes place via cooperative recognition by U11 and U12 of the di-snRNP, respectively 11. Minor introns lack a PPT and consequently do not require U2AF for intron recognition 3,29. Furthermore, the 3'ss is recognized by the Urp/ZRSR2 protein that possibly functions in both spliceosomes, albeit at different stages of the spliceosome assembly 30,31,32. The outcome of these differences is that the recognition of minor introns is somewhat more rigid and conservative compared to that of major introns.

The subsequent steps in the splicing process are very similar between the two systems. The formation of an initial intron recognition complex is followed by the association of a specific tri-snRNP with the nascent spliceosome. U1/5’ss or U11/5’ss interaction is replaced by U6/5’ss or U6atac/5’ss interaction, respectively, and dissociation of U1 or U11 snRNP from the pre-mRNA. Subsequently, the helices formed between U4/U6 or U4atac/U6atac are unwound, followed by the formation of the catalytic core composed of U2/U6 or U12/U6atac snRNAs base-paired to each other and to 5’ss and BPS sequences in the intron 10,33,34. This catalytic structure, together with associated proteins carries out the two-step splicing reaction which is identical between the two systems.

Although parallels can be drawn in the assembly and catalytic pathways of the two spliceosomes, the key question that remains unanswered is the reason for their co-existence. Present evidence suggests two salient functional differences at the level of whole mRNAs. First, unlike the U2-type introns that are subject to extensive alternative splicing processes that result in multiple mRNA isoforms from a single gene, only a handful of alternative splicing events have been described for U12-type introns 5,35,36. It is conceivable that the more rigid intron recognition process by the U11/U12 di-snRNP, coupled with the high conservation of the 5’ss and BPS sequences limits possibilities in alternative splice site selection. Second, both *in vitro* and *in vivo* investigations have provided evidence that minor intron splicing is less efficient than splicing of U2-type introns, trapping partially processed mRNAs containing unspliced U12-type introns in the nucleus 37,38,39,40,41. Such rate-limiting regulation of gene expression is then expected to downregulate the mRNA and protein levels of the genes containing U12-type introns, but at the same time this process may be further regulated by internal and external signals thereby creating an additional layer of gene expression control via minor intron splicing 42,43. Even though U12-type introns are located in a highly-conserved set of "host" genes, this group of genes does not constitute a simple group or discreet pathways. Rather, a more broad term of "information processing genes" was coined by Burge, Padgett and Sharp 3 to distinguish genes involved in DNA replication and repair, transcription, RNA processing and translation, cytoskeletal organization, vesicular transport, voltage-gated ion channel activity and Ras-raf signaling from those involved in basic metabolism 3,44,45.

## MUTATIONS OF MINOR SPLICEOSOME COMPONENTS AND NEURODEVELOPMENT DEFECTS

At present up to 60% of human diseases have been linked to splicing defects, with mutations in either components of spliceosomes or more commonly *cis*-acting regulatory elements within introns or exons and splice sites being the major contributors 46,47,48. Consequences and severity of these mutations depend on whether *trans*-acting splicing factors or *cis* elements are affected. Typically, mutations within genes encoding splicing factors tend to result in widespread defects as the function of entire splicing machinery can be compromised. Of the two systems, the major spliceosome has been studied at more detailed level and in fact, most diseases associated with splicing defects, both in *cis*-acting elements and in *trans*-acting factors, have been linked to major spliceosome function. These have been discussed in detail elsewhere 49,50. However, a rapidly growing body of knowledge and interest in the minor spliceosome in recent years has led to the discovery of a small number of diseases caused by mutations in core minor spliceosome components that could provide more insight into the significance of the minor spliceosome and explain the existence of two splicing machineries. Minor spliceosome-associated diseases have recently been reviewed 51 and as such only brief descriptions will be provided here. Presently, five congenital human diseases with defects in minor spliceosome components have been described: Microcephalic Osteodysplastic Primordial Dwarfism type I/Taybi-Linder Syndrome (MOPD1/TALS), Roifman syndrome (RFMN), Lowry Wood Syndrome (LWS), Early Onset Cerebellar Ataxia (EOCA), and Isolated Growth Hormone Deficiency (IGHD) with associated pituitary hypoplasia.

Three of the diseases, MOPD1/TALS, RFMN and LWS are autosomal recessive disorders which have been associated with point mutations in the *RNU4atac* locus, encoding the U4atac snRNA, an essential snRNA component in the U4atac/U6atac.U5 tri-snRNP. The patients amongst the three diseases are typically compound heterozygotes except for a single A51G>A mutation that as a homozygote leads to the most severe case of MOPD1/TALS 51,52,53,54,55,56. Inter-estingly, both LWS and RFMN patients are compound heterozygotes for U4atac mutations that are either shared with MOPD1/TALS or unique to LWS or RFMN. Clinically, all three diseases appear to overlap and share phenotypes related to cephalo-skeletal dysplasia, intrauterine and postnatal growth retardation and microcephaly, with varying severities 55,56,57. Additionally, MOPD1/TALS includes severe forms that are defined by general developmental defects in multiple organs and major brain malformations with death in infancy or childhood 52. In contrast, RFMN and LWS are phenotypically different and milder diseases but share the growth retardation and immune system (subclinical in LWS) defects of MOPD1/TALS. Additionally, both LWS and RFMN patients exhibit cognitive delays and facial dystrophies 54,55.

Recently, a mutation associated with EOCA was found in the *RNU12* gene which codes for the U12 snRNA that is part of the U11/U12 di-snRNP complex 58. Cerebellar ataxia is characterized by abnormal development and/or degeneration of the cerebellum. In the case of EOCA, patients homozygous for the mutation exhibit early (at infancy) muscle hypotonia, difficulties with speech and learning and abnormal gait 58. In connection with the U11/U12 di-snRNP, mutations in the *RNPC3* gene, encoding the U11/U12-65K protein, have been linked to IGHD, a genetically diverse disorder that is characterized by a deficiency or lack of growth hormone as a result of defective pituitary gland development. Moreover, IGHD patients with mutations in *RNPC3* also present a mild form of microcephaly 59,60.

## THE MINOR SPLICEOSOME AND NEURODEGENERATIVE DISEASES

Interestingly, while the small number of diseases linked to congenital mutations in minor spliceosome components show diverse and often pleiotropic pathologies, these diseases all share neurological components with varying degrees of severity. Similarly, several recent studies have also linked defects in the splicing of minor introns with neurodegenerative diseases such as Amyotrophic Lateral Sclerosis (ALS) and Spinal Muscular Atrophy (SMA), both of which are characterized by the degeneration of motor neurons. However, a feature that remains enigmatic is the tissue-specific phenotype observed in patients, regardless of the spliceosomal component or accessory factor affected. Here, we discuss the two currently known minor spliceosome-associated neurodegenerative diseases, emphasizing the points of convergence that illuminate the possible role and involvement of the minor spliceosome in cellular differentiation and function.

## SPINAL MUSCULAR ATROPHY

**Figure 2:**
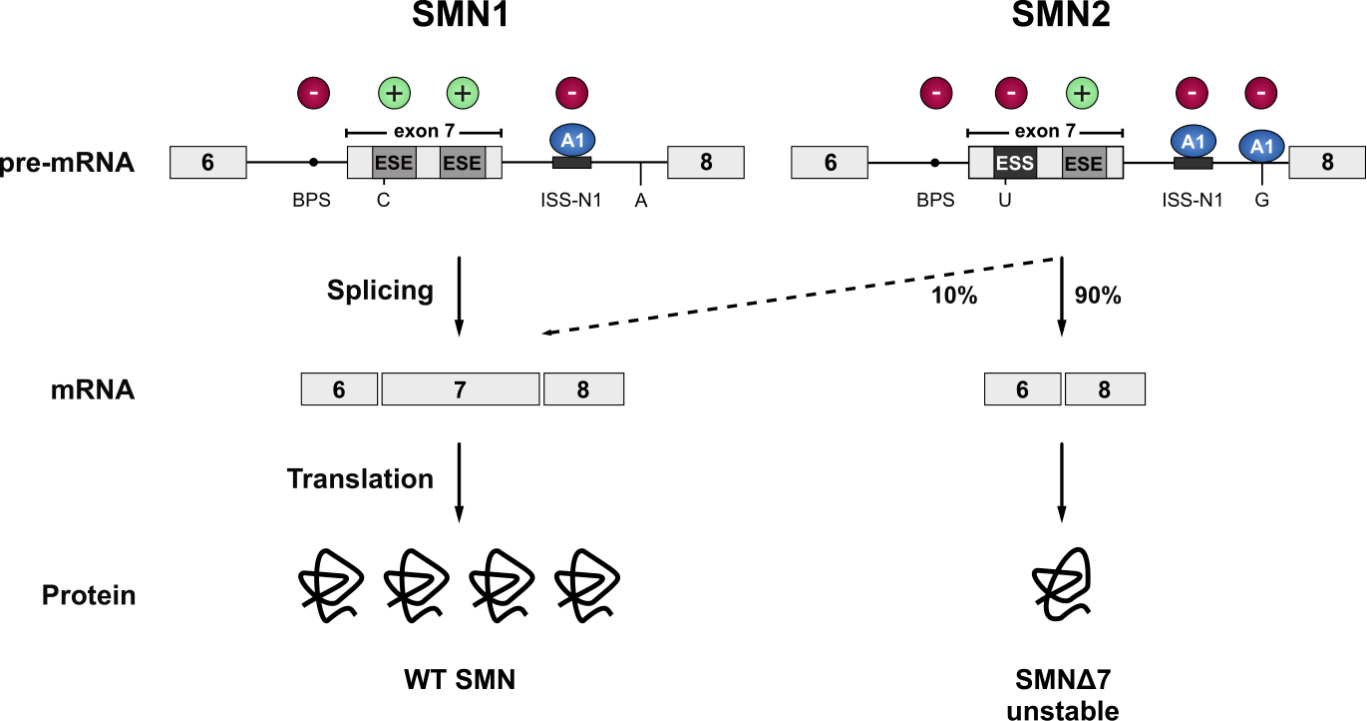
FIGURE 2: SMN1 and SMN2. The vast majority of the SMN protein is produced from the *SMN1* gene. However, during evolution, humans have acquired a paralogue (*SMN2*) by gene duplication, but *SMN2* produces only approximately 10% of the full-length mRNA. Due to a C to T transition in *SMN2* (C to U at RNA level) the first exonic splicing enhancer (ESE) in exon 7 is disrupted and an exonic splicing silencer (ESS) is created, which is bound by hnRNPA1 (A1). Inclusion of exon 7 is further prevented by an A to G transition further downstream in intron 7 (creating an additional hnRNPA1 binding site), and the suboptimal branchpoint (BPS) in intron 6. Hence, exon 7 is mainly skipped leading to the production of an instable C-terminally truncated protein (SMNΔ7) that is rapidly degraded. Cis-acting elements promoting exon inclusion are indicated with green plus signs, while inhibitory elements are marked with red minus signs.

Spinal Muscular Atrophy (SMA) is the most common motor neuron disease in children with an estimated incidence of 1 in 6’000 to 1 in 10’000 live births. Pathological hallmarks include the degeneration of motor neurons in the anterior horn of the spinal cord and brain stem and concomitant muscle atrophy 61,62. The disease is caused by decreased levels of the survival motorneuron (SMN) protein due to homozygous loss or mutation of the *SMN1 *gene 63. A complete loss of SMN protein is embryonic lethal 64,65. In SMA patients this lethality is rescued by a paraloguous *SMN2* gene that humans have acquired by gene duplication 66. However, *SMN2* harbours a silent C to T transition in exon 7 which disrupts an exonic splicing enhancer and converts it to an exonic splicing silencer 67,68. The combined effect of the suboptimal intron 6 branchpoint, the strong intronic splicing silencer in intron 7, and an A to G transition in *SMN2* further downstream in intron 7, that creates an hnRNPA1 binding site acting as an additional splicing silencer, result in a splicing pattern where exon 7 is predominantly skipped. This leads to the production of a C-terminally truncated protein that is rapidly degraded (**Figure 2**) 69,70,71,72. Thereby, *SMN2* produces only a fraction of full-length SMN mRNA, resulting in severely decreased SMN levels. The SMN protein is ubiquitously expressed and shows bimodal localization in both the cytoplasm and the nucleus, where it is enriched in biomolecular condensates termed Gemini of Cajal Bodies (GEMs) 63,73. These membrane-less compartments often physically associate with Cajal Bodies (CBs) which have been implicated in snRNP maturation and recycling 74. However, GEMs are not detected in all tissues, and are thus likely not essential for splicing, but are prevalent in cell types with high metabolic or transcriptional activity such as neurons 75. Although the function of GEMs remains enigmatic, their abundance is clearly linked to SMN levels and correlates with SMA disease severity 76. In neurons, SMN was also detected in axons and growth cones 77,78. The protein executes its various cellular functions as part of the macromolecular SMN complex composed of SMN, Gemins2-8 and Unrip. The best-characterized function of this 20S complex is the assembly of Sm-class snRNPs, by an ATP-dependent loading of a heptameric Sm-protein ring in the cytoplasm and their subsequent import into the nucleus. The Sm-class snRNPs either constitute the building blocks of both splicing machineries (U1, U2, U4, U5, U11, U12, U4atac) or play a critical role in the 3`-end processing of replication-dependent histone messenger RNAs (U7). In addition, SMN has been implicated in several other processes of the eukaryotic RNA metabolism, ranging from transcription 79, snoRNP and signal recognition particle (SRP) biogenesis 80,81, stress granule formation 82 and mRNA trafficking 77,78 to translation 83. However, these proposed functions are largely based on interaction data and currently lack rigorous biochemical validation.

## AMYOTROPHIC LATERAL SCLEROSIS

Amyotrophic Lateral Sclerosis is the most common motor neuron disease in adults with an estimated incidence of 1 - 3 per 100’000 and a prevalence of 3-7 per 100’000 indivi-duals worldwide 84. The primary symptoms of ALS are progressive muscle weakness, muscle atrophy and spasticity, which are caused by the degeneration and death of the upper (UMN) and lower (LMN) motor neurons in the motor cortex, the brainstem and spinal cord. The onset of ALS usually affects the limb-innervating motor neurons (spinal-onset). However, 20-25 % of ALS patients present a bulbar onset. In 3-5 % of the cases, the disease starts with initial trunk or respiratory involvement 85,86. However, there are also less common cases of exclusive UMN or LMN de-generation reported. The degeneration of the UMNs results in speech problems (especially in bulbar onset), weakness, spasticity and uncontrollable reflexes, whereas the loss of LMNs results in muscle wasting and weakness as well as in cramps and decreased reflexes 85,87,88,89,90. The average disease onset peaks between 50-75 years of age, and death due to respiratory failure typically occurs within 2 - 5 years after the first symptoms 91. However, there is a large variability among patients, and disease progression and survival can vary from months to decades. The observation that family members that harbour identical ALS-causing mutations show differences in disease onset and survival, implicates the presence of genetic or environmental disease modifiers that affect severity and progression rate 92. Additionally, recent work has revealed that ALS represents a disease spectrum together with frontotemporal dementia (FTD) 93. The most striking support for the spectrum disease concept arose from large genetic and histopathological overlaps as identical mutations are present in families with either ALS, FTD or both diseases 94,95,96. FTD is one of the most common forms of dementia in patients younger than 65 years and is characterized by atrophy of the temporal and frontal brain lobe and by behavioural changes or speech impairment 97,98,99,100. Clinically, depending on the population studied, 10-50% of ALS patients develop some symptoms of FTD and 10-15% of patients with FTD show symptoms of motor neuron disease 101,102,103,104,105,106.

While most ALS patients suffer from the apparent sporadic form of the disease (sALS), about 10 % of the patients have clearly inherited the disease 107. These ALS cases (fALS), which clinically and pathologically are indistinguishable from the sporadic form, frequently show either point mutations in the genes coding Cu/Zn-binding superoxide dismutase 1 protein (SOD1) 108, the TAR DNA binding protein 43 (TDP-43) 96,109,110, the RNA-binding protein Fused in Sarcoma (FUS) 111,112 or hexanucleotide expansions in the first intron of the *C9orf72* gene 113,114. The mutations in these four genes account for approximately 55% of the familial ALS cases (fALS) but are also found in sporadic ALS cases (sALS), albeit to a much lesser extent 115. Unfortunately, the causes for the vast majority of sALS cases remain unknown. It has been proposed that susceptibility is increased by an interplay of low penetrance genetic risk factors, exposure to environmental risk factors, and subsequent accumulation of cell damage with age 116,117. Mutations in many different genes have been found to be causative for fALS thereby explaining 68% of fALS cases 117. However, the connection between these mutated genes and development of ALS remains elusive. A converging feature in ALS is the presence of ubiquitinated cytoplasmic inclusions in the degenerating motor neurons and glia cells. With the notable exception of *FUS* and *SOD1* mutations, which cause aggregation of FUS and SOD1, respectively, most ALS cases display ubiquitinated TDP-43 inclusions 110. C9orf72-linked ALS also presents, apart from TDP-43 pathology, intranuclear RNA foci as well as ubiquitin reactive and TDP-43-negative inclusions in the cerebellum and the hippocampus. These contain dipeptide repeat proteins translated from the hexanucleotide repeats 113,118,119.

## A ROLE FOR THE MINOR SPLICEOSOME IN SMA AND ALS?

ALS and SMA are both neurodegenerative diseases that lead to the loss of motor neurons and consequently voluntary muscle movement. Despite the differences in age of onset, disease progression and etiology, FUS-linked and TDP-43-linked ALS and SMA converge with each other 120,121, and with the minor spliceosome. Of the two diseases, SMA provides a more direct link to the minor spliceosome. Even though the SMN mutations in SMA are expected to impair the assembly of both major and minor Sm-class snRNPs, several reports suggest that this effect may be exacerbated with the minor spliceosome components. Specifically, investigations of the spinal cord and brain of moderate and severe SMA mouse models have revealed preferential downregulation of the minor spliceosome snRNPs 122,123. This effect was not observed in tissues unaffected by the disease, which further supports the hypothesis that decreased snRNP levels are directly involved in the SMA pathomechanism 123. Additionally**,** minor tri-snRNP formation is impaired in SMA patient-derived lymphoblasts 124 and widespread mis-splicing of U12-type introns was observed not only in SMA patient-derived cells but also in Drosophila and Mouse SMA models 124,125,126.

In the context of ALS, depletion of TDP-43 has a direct effect on the minor spliceosome components with various minor snRNAs being misregulated in a cell-line specific manner. In SH-SY5Y cells the levels of U4atac and U6atac snRNAs are reduced by depletion of TDP-43, while in U87MG cells the levels of U12 snRNA are reduced and U11 are increased. In contrast, HeLa cells show no significant changes in the level of minor spliceosome in response to TDP-43 depletion 127. Likewise, sALS patients with TDP-43 pathology show a misregulation of minor snRNAs in their spinal cord, motor cortex and thalamus compared to control patients 127,128. Additionally, reduced nuclear levels of the 59K protein subunit of the U11 snRNP were reported in spinal cord motor neurons 127. A possible connection between the minor spliceosome and TDP-43 are the GEMs, as suggested by colocalization of TDP-43 and GEMs in HeLa cells, SH-SY5Y cells and mice hippocampal neurons 128. Consistently, knockdown of TDP-43 in HeLa cells reduces the number and size of GEMs 127,128, while mice with a conditional TDP-43 knock out failed to form GEMs altogether in upper motor neurons 129. This combined evidence suggests a role for TDP-43 in GEM formation. Intriguingly, compared to other cells, motor neurons stand out with the highest density and increased size of GEMs 75, which makes them particularly interesting not only in the context of SMA but also ALS.

Similar observations were made with FUS. Primary cultured hippocampal neurons from knock-out FUS mice and HeLa cells with a FUS knock-down failed to form GEMs 128,130,131,132. Furthermore, FUS directly interacts with SMN 128,132,133 and fibroblasts of ALS patients harboring a mutation in the NLS of FUS (R521C and R514G) show a reduced number of GEMs 132. Finally, it was shown that FUS interacts with spliceosomal snRNAs and that cytoplasmic FUS inclusions specifically trap snRNAs, thereby decreasing their nuclear concentration 132,134,135, thus suggesting converging pathomechanisms via decrease of spliceosomal snRNPs in the nucleus. However, how could disturbance of such a general function as splicing confer selective motor neuron death as observed in FUS- and TDP-43-linked ALS and SMA? One possible explanation is a selective or preferential impairment of minor intron splicing.

While a direct function of TDP-43 in minor intron splicing remains to be elucidated, FUS preferentially interacts with minor intron containing mRNAs and with the minor spliceosome 135. FUS depletion affects over 30% of minor spliceosome-dependent splicing events and leads to extensive downregulation of minor intron containing genes involved in neuronal functions, such as promotion of neurogenesis, dendritic development, postnatal maturation of spinal motor units and axonal outgrowth. Additionally, ALS-linked FUS is splicing insufficient as it localizes to the cytoplasm and therefore cannot participate in splicing of the nuclear pre-mRNAs 135. Furthermore, cytoplasmic FUS inclusions trap significant amounts of minor snRNAs in the cytoplasm leading to apparent nuclear reduction implicating a general minor spliceosome defect in FUS-linked ALS 135. Interestingly, C9orf72-linked ALS cases not only display TDP-43 pathology but the expressed hexanucleotide repeat expansion RNA also sequesters hnRNP H 113,136. HnRNP H participates in the splice site recognition of many minor introns, and is involved in the autoregulation of the U11-48K protein that is essential for the 5'ss recognition of minor introns 137,138,139. Furthermore, hnRNP H seems to require FUS to efficiently promote splicing of a subset of transcripts 135, suggesting that misregulation of hnRNP H in C9orf72-linked ALS may be an additional factor promoting mis-splicing of minor introns.

**Figure 3:**
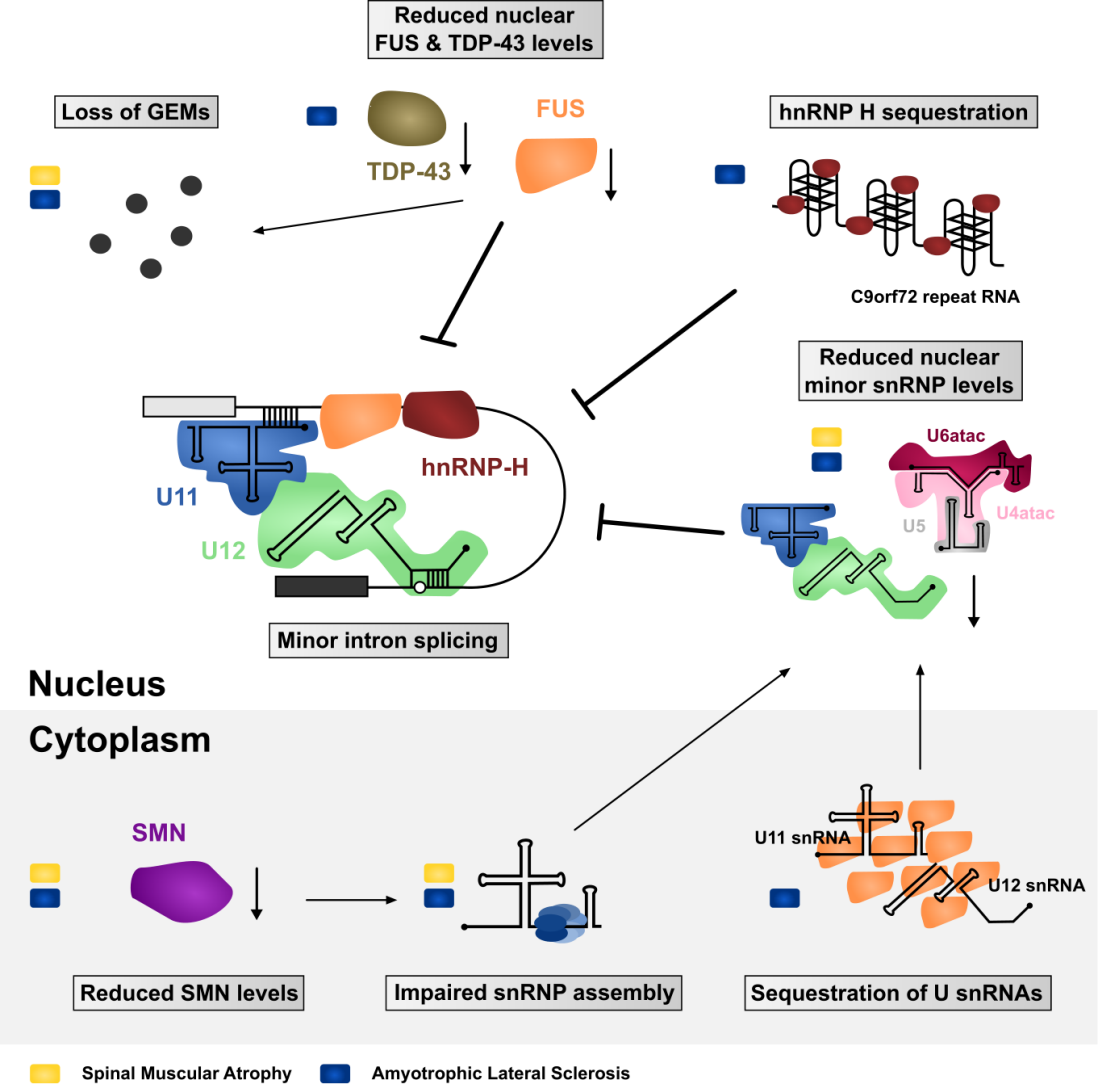
FIGURE 3: Impaired minor intron splicing in SMA and ALS. Many of the molecular defects observed in patient tissues or disease modelling systems ultimately converge on affecting the spliceosome and minor intron splicing. These include both gain- and loss-of-function mechanisms in the nucleus as well as in the cytoplasm. Defects linked with SMA are indicated with a yellow rectangle, while blue rectangles mark ALS-associated defects.

Together, SMA and ALS with FUS/TDP-43 pathology have both been associated with defects in minor intron splicing and/or abnormal cellular distribution of minor spliceosome components (**Summarized in Figure 3**). But is there evidence that further links such defects to motor neuron pathology? In contrast to major introns, minor introns are neither present in housekeeping genes nor evenly distributed throughout the genome but mainly present in genes related to information processing 3,4,45. Some of these minor intron containing genes fulfil crucial roles for maintenance of neuromuscular junctions which are primary pathological targets in ALS and SMA, whereas other genes are required for general motor neuron function 126,135,140,141,142,143,144,145. For example, SMA mice display in-creased minor intron retention and concomitant downregulation of functional mRNA from the *Myo10* gene encoding a member of the myosin-family of motor proteins that has been associated with axon outgrowth and neuronal development 146,147. Similarly, the minor intron containing *Stasimon* gene (*TMEM41b* in humans), which is required for motor neuron development and function has been reported to be aberrantly spliced in Drosophila, Zebrafish, and Mouse models of SMA 125,126. Strikingly, injection of human TMEM41b mRNA is sufficient to rescue neuromuscular junction transmission defects caused by decreased SMN levels in these model systems 126. Finally, a number of minor intron containing genes are known to code for voltage-gated ion channels that are necessary for motor neuron function 45,143,148,149. Among them are, for example, several subunits of voltage-gated calcium channels that are affected in SMA: *Cacna1a, Cacna1b, Cacna1c, Cacna1e *and* Cacna1h *125. Intriguingly, some of these genes are also affected by FUS depletion in agreement with reported disruption of Ca^2+^ homeostasis and axonal defects in both SMA and ALS 150,151,152,153. Finally, as proposed by Doktor *et al*. 125, SMA can be modelled in evolutionary distinct organisms, which suggests that the underlying defect is evolutionarily conserved. A similar argument can also be made for ALS 154. Therefore, it is possible that while major spliceosome defects can contribute to specific phenotypes, the high degree of conservation of minor introns could explain the common pathology across species and over large evolutionary distances.

However, while there is growing evidence for a contribution of the minor spliceosome to the pathomechanism of neurodegenerative disorders, most of the data are correlative and the specific mechanism or the other contributions are not yet fully elucidated. For example, SMN, FUS and TDP-43 all have other axonal functions that are impaired in SMA and ALS, such as control of genes that are involved in neuritogenesis and axonal outgrowth that could further contribute to the neurodegenerative phenotype 155,156,157,158,159. Therefore, the future challenge is to determine the specific contribution of the minor spliceosome in neurodegeneration. For example, whether the defects or alteration in minor intron splicing contribute directly to neurodegeneration via mis-splicing of specific neuronal or muscular genes affecting neuronal survival or via maintenance of neuromuscular junctions. Due to the rather low number of minor introns in the genome, a comparative transcriptomic study of ALS and SMA motor neurons in isolation or NMJs from the same species with identical genetic background should reveal whether a defined set of shared mRNA-processing events in specific genes lead to neurodegeneration. While such a candidate gene hypothesis is highly appealing, an alternative possibility is that a global and possibly mild defect in the splicing of minor introns may affect the expression of hundreds of genes, the combined effect of which subsequently compromises neuronal survival. Minor introns are found in genes responsible for DNA repair, RNA processing, cytoskeletal organization, and neuronal transmission and indeed all these processes have been found to be affected both in ALS and SMA 92,160,161,162. Furthermore, minor spliceosome components are of low abundance already in proliferating cells and at least a subset of them are further downregulated during neuronal differentiation 163. Hence, while other cell types might tolerate the down-regulation of minor intron splicing activity due to reduction or partial sequestration of minor spliceosome components, neuronal cells might be particularly vulnerable towards these disturbances after differentiation. Thus, the accumulation of small but global alterations in the expression of genes containing minor introns may add up and lead to reduced survival of neurons or their dysfunction.

## Abbreviations

3‘ss: 3’ splice site
5‘ss: 5’ splice site
ALS: Amyotrophic Lateral Sclerosis
BPS: branch point sequence
EOCA: Early Onset Cerebellar Atrophy
fALS: familial ALS
FTD: frontotemporal dementia
GEM: Gemini of Cajal bodies
IGDH: Isolated Growth Hormone Deficiency
LMN: lower motor neuron
LWS: Lowry Wood Syndrome
MODPD1/TALS: Microcephalic Osteodysplastic Primordial Dwarfism type 1 / Taybi-Linder syndrome
RFMN: Roifman syndrome
sALS: sporadic ALS
SMA: Spinal Muscular Atrophy
snRNA: small nuclear RNA
snRNP: small nuclear ribonucleoprotein
UMN: upper motor neuron
